# Alantolactone alleviates collagen-induced arthritis and inhibits Th17 cell differentiation through modulation of STAT3 signalling

**DOI:** 10.1080/13880209.2021.1876102

**Published:** 2021-02-08

**Authors:** Hsiang-Lai Chen, Shih Chao Lin, Shiming Li, Kuo-Tung Tang, Chi-Chien Lin

**Affiliations:** a Department of Surgery, Division of Urology, Tung's Taichung MetroHarbor Hospital, Taichung, Taiwan, ROC; b Program in Translational Medicine, National Chung Hsing University, Taichung, Taiwan, ROC; c Bachelor Degree Program in Marine Biotechnology, College of Life Sciences, National Taiwan Ocean University, Keelung, Taiwan, ROC; d College of Chemistry & Chemical Engineering, Hubei Key Laboratory for Processing & Application of Catalytic Materials, Huanggang Normal University, Huanggang, PR China; e Faculty of Medicine, National Yang-Ming University, Taipei, Taiwan, ROC; f Division of Allergy, Immunology and Rheumatology, Taichung Veterans General Hospital, Taichung, Taiwan, ROC; g Institute of Biomedical Science, The iEGG and Animal Biotechnology Center, National Chung-Hsing University, Taichung, Taiwan, ROC; h Department of Medical Research, China Medical University Hospital, Taichung, Taiwan, ROC; i Department of Medical Research, Taichung Veterans General Hospital, Taichung, Taiwan, ROC; j Department of Pharmacology, College of Medicine, Kaohsiung Medical University, Kaohsiung, Taiwan, ROC

**Keywords:** *Inula helenium* L., RORγt, synovial hyperplasia, bone erosion, IL-6, IL-17A, anti-inflammation

## Abstract

**Context:**

Alantolactone, the bioactive component in *Inula helenium* L. (Asteraceae), exhibits multiple biological effects.

**Objective:**

We aimed to determine the anti-inflammatory effect of alantolactone in a collagen-induced arthritis (CIA) mouse model and its immunomodulatory effects on Th17 differentiation.

**Materials and methods:**

A CIA mouse model was established with DBA/1 mice randomly divided into four groups (*n* = 6): healthy, vehicle and two alantolactone-treated groups (25 or 50 mg/kg), followed by oral administration of alantolactone to mice for 21 consecutive days after arthritis onset. The severity of CIA was evaluated by an arthritic scoring system and histopathological examination. Levels of cytokines and anti-CII antibodies as well as percentages of splenic Th17 and Th17 differentiation with or without alantolactone treatments (0.62, 1.2 or 2.5 μM) were detected with ELISA and flow cytometry, respectively. Western blot analysis was used to evaluate intracellular signalling in alantolactone-treated spleen cells.

**Results:**

In CIA mice, alantolactone at 50 mg/kg attenuated RA symptoms, including high arthritis scores, infiltrating inflammatory cells, synovial hyperplasia, bone erosion and levels of the proinflammatory cytokines TNF-α, IL-6 and IL-17A, but not IL-10 in paw tissues. Alantolactone also reduced the number of splenic Th17 cells and the capability of naïve CD4^+^ T cells to differentiate into the Th17 subset by downregulating STAT3/RORγt signalling by as early as 24 h of treatment.

**Discussion and conclusions:**

Alantolactone possesses an anti-inflammatory effect that suppresses murine CIA by inhibiting Th17 cell differentiation, suggesting alantolactone is an adjunctive therapeutic candidate to treat rheumatoid arthritis.

## Introduction

Rheumatoid arthritis (RA) is an autoimmune disease caused by the production of autoantibodies and joint inflammation (Smolen et al. 2018). Its main characteristics are chronic synovial inflammation, leading to the destruction and erosion of joints and ultimately the loss of joint functions (Chehata et al. 2001; McInnes and Schett 2011). Approximately, 1% of the population suffers from RA worldwide. Patients have a poor quality of life, and the resulting disability affects their physical functions and even life expectancy (Chehata et al. 2001; McInnes and Schett 2011). Current medical therapies for RA, including disease-modulating anti-rheumatic drugs (DMARDs) and biologics, effectively ameliorate joint inflammation. However, side effects, such as infection and hepatotoxicity, are often detrimental (Caporali et al. 2008; Aithal 2011; Kourbeti et al. 2014). The development of therapeutics for RA with fewer side effects is urgent.

The pathogenesis of RA is elusive. Recently, dysregulated Th17 cells were reported to be one of the RA pathogenic pathways (Yang et al. 2014). Th17 cells are highly associated with autoimmune diseases, such as RA, systemic lupus erythematosus and multiple sclerosis (Yang et al. 2014; Roeleveld and Koenders 2015; Dos Passos et al. 2016; Alvarez-Rodriguez et al. 2019). Th17 cells secrete IL-17 (Kimura et al. 2007), which is implicated in joint inflammation and bone erosion in RA (Ziolkowska et al. 2000; Parsonage et al. 2008; Zrioual et al. 2009; Kim et al. 2015). In a collagen-induced arthritis (CIA) mouse model, neutralizing IL-17 antibody can significantly alleviate arthritis severity (Kelchtermans et al. 2009). Clinical trials also supported some efficacy of anti-IL-17 agents against RA without many adverse effects (Kunwar et al. 2016). Th17 differentiation depends on the transcription factor RORγt. The expression of RORγt is regulated by both interleukin (IL)-6 and transforming growth factor (TGF)-β (Bettelli et al. 2006; Veldhoen et al. 2006). IL-6 can activate signal transducer and activator of transcription 3 (STAT3), which induces RORγt expression and thereby promotes Th17 differentiation (Zhou et al. 2007; Chang et al. 2020). TGF-β inhibits the expression of suppressor of cytokine signalling 3 (SOCS3), a negative regulator of STAT3, and further promotes IL-6/STAT3/RORγt signalling and Th17 polarization (Qin et al. 2009). Based on these findings, we speculated that the regulation of STAT3 signalling could be a drug target to inhibit pathogenic Th17 differentiation in RA.

Certain plant compounds have been found to be beneficial for human health, especially those with antioxidant properties (Crozier et al. 2009; Martin et al. 2011). Recent studies have taken advantage of the immune regulatory characteristics of these plant compounds to treat inflammatory diseases (Rios et al. 2009), such as asthma, cardiovascular disease and RA (Teixeira Damasceno et al. 2007; Morinobu et al. 2008; Gonzalez-Gallego et al. 2010; Liu 2013). Historically, roots of *Inula helenium* L. (Asteraceae) are used in Chinese medicine to treat gastroenteritis and bronchitis (Seca et al. 2014; Gierlikowska et al. 2020). The bioactive components in *Inula helenium* extracts consist of sesquiterpene lactones, and the major components are alantolactone and isoalantolactone (Wang, Gao, et al. 2018). *In vitro* and *in vivo* studies have reported their antibacterial, antifungal, anticancer and anti-inflammatory effects (Cantrell et al. 1999; Stojanovic-Radic et al. 2012; Liu et al. 2018; Tan et al. 2019), partly through the inhibition of the STAT3 signalling pathway (Khan et al. 2013; Kim et al. 2017; Maryam et al. 2017; Zheng et al. 2019). No studies have reported the effects of these components on Th17 cell differentiation in autoimmune diseases.

Here, we used IL-6 and TGF-β to induce mouse splenic T cells *in vitro* to differentiate into Th17 cells and explored the effect of alantolactone on Th17 differentiation. We also used a murine model of CIA to evaluate its *in vivo* effects on Th17 cell differentiation and joint inflammation.

## Materials and methods

### Animals

Eight-week-old female DBA/1 mice were obtained from the Jackson Laboratory (Bar Harbor, ME), and male C57BL/6 mice were purchased from the National Laboratory Animal Center (Taipei, Taiwan). All mice were kept in rooms under controlled temperature, humidity and light (12 h light–dark cycle) with water and food *ad libitum*.

All experimental protocols were conducted according to our institutional guidelines with approval from the Institutional Animal Care and Utilization Committee of National Chung Hsing University, Taiwan (approval number NCHU-IACUC-109-086).

### Isolation of naïve CD4 T cells and *in vitro* induction of Th17 cell differentiation

CD4 T cells were positively enriched from splenocytes using EasySep Murine CD4 T cell selection kits (Stem Cell, Grenoble, France) according to the manufacturer’s instructions. The purity of CD4 T cells was >90%, as determined by flow cytometry with FITC-conjugated anti-CD4 monoclonal antibodies (mAbs). The purified CD4 T cells were cultured in 12-well plates containing plate-bound anti-CD3 (1 µg/mL, BioLegend, Inc., San Diego, CA) and soluble anti-CD28 (1 μg/mL, BioLegend, Inc., San Diego, CA) at 2 × 10^6^ cells/well in RPMI 1640 medium (Invitrogen, Rockville, MD) supplemented with 10% FBS, 100 U/mL penicillin and 100 mg/mL streptomycin in a humidified incubator at 37 °C and 5% CO_2_.

**Figure 1.**
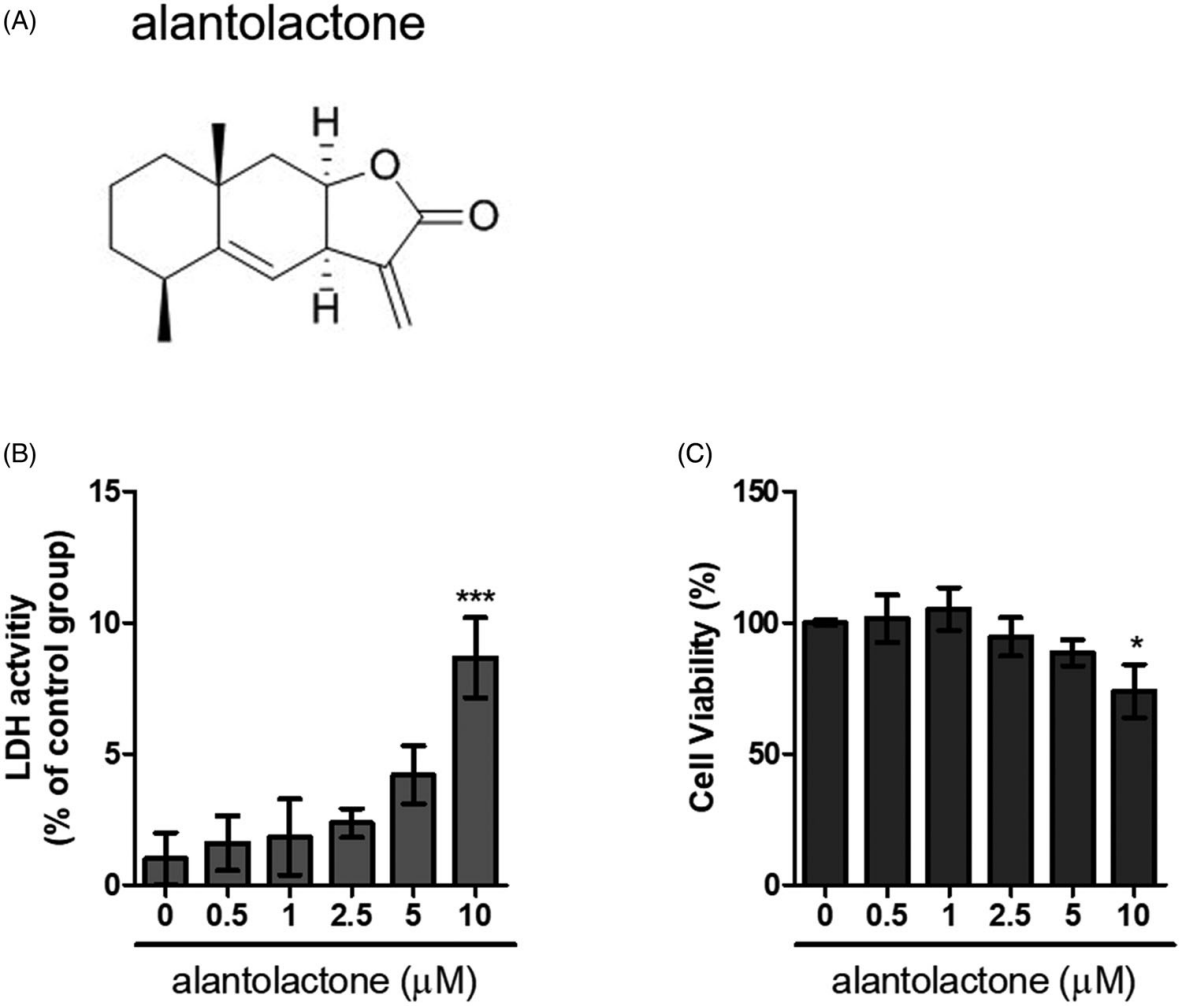
Cytotoxicity of alantolactone against CD4 T cells. (A) Chemical structure of alantolactone, (B) LDH and (C) CCK-8 assays were conducted to measure cytotoxicity after incubating CD4 T cells with various concentrations of alantolactone for 72 h. Data are representative of three independent experiments, and values are expressed as the mean ± SEM of samples of three wells. *p < 0.05, ***p < 0.001 compared with untreated controls, based on one-way ANOVA with Dunnett’s test.

For the Th17 cell polarization experiment, we adopted the protocol reported in the literature (Veldhoen et al. 2006; Stockinger and Veldhoen 2007). In brief, CD4 T cells were cultured for 72 h with anti-IL-4 (10 μg/mL, clone 11B11, BioLegend, San Diego, CA), anti-IFN-γ (10 μg/mL, BioLegend, San Diego, CA), TGF-β (2.5 ng/mL, Peprotech, Rocky Hill, NJ) and IL-6 (20 ng/mL, PeproTech, Rocky Hill) antibodies. Half of the culture medium was replaced by medium containing fresh antibodies. Alantolactone (>98%, molecular weight of 232.31, ChemFaces, Wuhan, China; Figure 1(A)) was dissolved in dimethyl sulphoxide (DMSO, Sigma, St. Louis, MO). Cells were incubated with different concentrations of alantolactone for 72 h. In the control groups, DMSO was used at amounts similar to those in the treatment groups.

### Lactate dehydrogenase (LDH) assay

An LDH cytotoxicity assay kit (Cayman Chemical Co., Ann Arbor, MI) was employed to assess the cellular toxicity induced by the compounds. CD4 T cells were plated in 96-well plates (5 × 10^4^ cells/well) prior to incubation for 72 h at different concentrations of alantolactone. Cells were then incubated with Triton X-100 and centrifuged at 1200 rpm for 5 min before collecting the supernatant. The supernatant (100 µL/well) was transferred to a new 96-well plate in which an equal volume of LDH reagent was added to each well. After incubation for 30 min at room temperature, LDH concentrations were measured at a wavelength of 490 nm on a microplate reader (TECAN, Durham, NC). The level of LDH released (% of positive control) was recorded as a percentage of (ODtest – ODblank)/(ODpositive – ODblank), where ODtest was the optical density of cells treated with either 0.1% DMSO or alantolactone; ODpositive was the optical density of 1% Triton X-100-treated cells and ODblank was the background optical density of the wells without cells.

### CCK-8 cell viability assay

CD4 T cells were incubated in 96-well plates (5 × 10^4^/well) for 72 h at different concentrations of alantolactone. Then, reagent (10 μL) from Cell Counting Kit-8 (CCK-8) (Dojin Laboratory Co., Kumamoto, Japan) was added to the wells, and the plates were incubated for 4 h before the absorbance intensity of the formazan product was determined at 450 nm.

### RNA isolation and real-time PCR

CD4 T cells were incubated in 96-well plates (5 × 10^4^/well) for 72 h under Th17-polarizing conditions with or without alantolactone. Total RNA was prepared from stimulated CD4 T cells using TRIzol reagent (Invitrogen, Rockville, MD). cDNA was synthesized for each RNA sample with a Superscript III Reverse Transcriptase system (Invitrogen, Carlsbad, CA). Real-time quantitative gene expression was determined with the AB 7500 Fast System (Applied Biosystems, Foster City, CA) using SYBR Green Master Mix (Roche, Basel, Switzerland). The 2^–ΔΔCT^ method was used to normalize transcription against GAPDH, and induction relative to controls was calculated as fold changes. Table 1 shows the primer pair sequences used for real-time PCR.

**Table 1.** Mouse gene PCR primer sequence.

Gene	Forward	Reverse
RORγδT	CCGCTGAGAGGGCTTCAC	TGCAGGAGTAGGCCACATTACA
IL-17A	CTCCAGAAGGCCCTCAGACTAC	AGCTTTCCCTCCGCATTGACACAG
GAPDH	CGTGTTCCTACCCCCAATGT	TGTCATCATACTTGGCAGGTTTCT

### Cytokine production

CD4 T cells were incubated in 96-well plates (5 × 10^4^/well) for 72 h under Th17-polarizing conditions with or without alantolactone. IL-17A concentrations were determined with mouse IL-17A ELISA kits according to the manufacturer’s instructions (eBioscience, San Diego, CA).

### Flow cytometry analysis

IL-17A produced by CD4 T cells affects the differentiation of Th17 cells. CD4 T cells (5 × 10^4^ cells/well) incubated for 72 h in 96-well plates under Th17-polarizing conditions were treated with or without alantolactone. During the last 4 h, we added phorbol-12-myristate-13-acetate (PMA, 100 ng/mL, Sigma-Aldrich, St. Louis, MO)/ionomycin (500 ng/mL, Sigma-Aldrich, St. Louis, MO) and GolgiStop (BD Bioscience, San Jose, CA) before cell harvest for surface staining with PerCP/Cyanine 5.5-conjugated anti-CD4 antibody (clone GK1.5, BioLegend, San Diego, CA). Cells were further stained intracellularly with FITC-conjugated anti-IL-17 antibody (clone TC11-18H10.1; BioLegend, San Diego, CA) using a Cytofix/Cytoperm Plus kit (BD Biosciences, San Jose, CA). The percentage of IL-17 + CD4 T cells was estimated on an Accuri 5 flow cytometer, and the mean fluorescence intensity was determined using C6 Accuri system software (Accuri Cytometer, BD Biosciences, San Jose, CA).

### Western blot analyses

CD4 T cells were incubated on 96-well plates (5 × 10^4^/well) for 24, 48 or 72 h under Th17-polarizing conditions with or without alantolactone. To prepare cell extract samples, cells were lysed in RIPA lysis buffer (EMD Millipore, Merck KGaA, Darmstadt, Germany). Protein concentrations were determined using the BCA Protein Assay Reagent (Thermo Fisher Scientific, Waltham, MA). Each sample (50 μg protein) was separated electrophoretically on an SDS-PAGE gel before transfer to a polyvinylidene difluoride (PVDF) membrane (Millipore, Bedford, MA). The membrane was blocked with 5% non-fat milk in TBST for 1 h at room temperature and then probed at 4 °C overnight with the following antibodies: anti-phospho-STAT3 (Tyr705) (clone 13A31, BioLegend, San Diego, CA), anti-STAT3 (clone 4G4B45, BioLegend, San Diego, CA), anti-phospho-JAK2 (Tyr1007, polyclonal, ab195055, Abcam, Cambridge, UK), anti-JAK2 (clone C-10, Santa Cruz Biotechnology, Santa Cruz, CA), anti-GAPDH (clone W17079A, BioLegend, San Diego, CA) or anti-RORγt (RORg2, BioLegend, San Diego, CA). After washing, membranes were exposed to horseradish peroxidase (HRP)-conjugated secondary antibodies (Jackson ImmunoResearch Laboratories, Inc., West Grove, PA) at room temperature for 2 h. The signals of the blots were developed using the enhanced chemiluminescence system (Perkin Elmer, Waltham, MA), and signals were detected using the Hansor Luminescence Image System (Taichung, Taiwan). Densities of target bands were estimated using ImageJ software (National Institute of Health, Bethesda, MD). The results were normalized against the corresponding GAPDH and normal control levels.

### CIA induction and alantolactone treatment

CIA was performed as described in the literature (Brand et al. 2007) but with only minor modifications. Briefly, 2 mg/mL bovine type II collagen (CII, Chondrex, Redmond, WA) was dissolved in 10 mM acetic acid and emulsified at a 1:1 ratio with complete Freund’s adjuvant (CFA, Chondrex, Redmond, WA). At the beginning of the experiments (day 0), the DBA/1 mice were intradermally injected with 0.2 mL of emulsion at their tail base, and on day 21, a booster injection was given with CII emulsified at 1:1 with incomplete Freund’s adjuvant (IFA; Chondrex, Redmond, WA). Alantolactone was dissolved in a solution of 10% DMSO and 90% glyceryl trioctanoate. Mice were randomly divided into four groups (six mice/group): normal, CIA, vehicle-treated CIA and alantolactone-treated CIA at concentrations of 25 or 50 mg/kg. Alantolactone was orally administered once daily from day 21 to day 42 after the first immunization. Control mice received only the solvent vehicle.

### Evaluation of arthritis severity in CIA

Clinical symptoms of arthritis were evaluated by two independent observers visually on individual limbs of the animals and graded on a scale of 0–4 in a blinded manner with a semiquantitative scoring system as described previously (Brand et al. 2007). Zero represented no erythema or swelling, and the maximum score of 4 indicated the most severe arthritis. The arthritis score for each animal was the sum of its four limbs. The sum of 16 was the maximal score.

### Histological examination

On day 42, mice were sacrificed by excessive CO_2_ inhalation. Joint tissues were removed from the hind paws followed by fixation with 4% paraformaldehyde, decalcification in 5% formic acid, and paraffin embedding. Paraffin sections obtained at 5 μm thickness were stained with haematoxylin and eosin (H&E) to reveal tissue structures. Histopathological changes, such as hyperplasia, cell infiltration and cartilage destruction in synovial tissues, were blindly assessed by a pathologist. A scoring system from 0 to 4 (Deng et al. 2005) was used: 0, no change; 1, mild change; 2, moderate change; 3, severe change; and 4, total destruction of joint architecture.

### Determination of anti-CII IgG antibodies

Serum samples collected from each mouse 42 days after the first immunization were used to determine anti-CII IgG antibody titres using ELISA. In brief, 96-well ELISA microtiter plates (Thermo Fisher Scientific, New York, NY) were first coated with 200 μL of type II bovine collagen (Chondrex, Redmond, WA) and kept at 4 °C overnight. Non-specific binding was blocked with PBS/3% bovine serum albumin (Millipore Sigma, St. Louis, MO) at 37 °C for an hour and then washed three times. Test sera were diluted 2500-fold, added to the well and kept at 4 °C overnight. After incubation, plates were washed 5–7 times with PBS/0.2% Tween-20 and incubated with 1:5000 HRP-conjugated sheep anti-mouse IgG (Jackson ImmunoResearch Laboratories, Inc., West Grove, PA) at 4 °C overnight. After thorough washing, ABTS colorimetric substrates (eBioscience, San Diego, CA) were added to the plates. Reactions were stopped by adding H_2_SO_4_, and the absorbance levels were measured at 450 nm with a microplate reader (TECAN, Durham, NC).

### Spleen cell proliferation and cytokine production after CII stimulation

On day 42, cell suspensions from the spleen were cultured in 96-well plates at 5 × 10^5^ cells/well with 200 μL of 10% foetal calf serum, 50 μg/mL gentamicin, 2 mM glutamine and 50 μM 2-mercaptoethanol. Cell suspensions were then stimulated with bovine CII (50 μg/mL) at 37 °C for 72 h. Spleen cell proliferation was quantified by incorporating [^3^H]thymidine (1 μL Ci/well, Amersham Pharmacia Biotech, Piscataway, NJ) during the last 18 h of culture. For the intracellular detection of cytokines, spleen cells were incubated with collagen for 48 h. GolgiStop (BD Biosciences, San Diego, CA) solution was added 6 h before cell harvesting. Cells were then washed twice in FACScan buffer and stained against the PerCP/Cyanine 5.5-conjugated anti-CD4 antibody (clone GK1.5, BioLegend, San Diego, CA). Splenocytes were fixed and subjected to intracellular staining using the Cytofix/Cytoperm Plus Kit (BD Biosciences, San Diego, CA) according to the manufacturer's instructions (BD Biosciences, San Diego, CA). FITC-conjugated mAbs specific to murine IL-17A were purchased from BioLegend (San Diego, CA, clone TC11-18H10.1). All samples were detected on an Accuri C5 cytometer using C6 Accuri system software (Accuri Cytometers Inc., Ann Arbor, MI).

### Cytokine measurements in paw tissues

Paw tissues were obtained on day 42. Frozen tissues (100 mg/sample) were homogenized with 1 mL of tissue lysis buffer containing the protease inhibitor cocktail (UltraCruz^®^, Santa Cruz Biotechnology, Santa Cruz, CA). Total protein concentrations were then determined using a bicinchoninic acid protein assay kit (Thermo Fisher Scientific, Waltham, MA). The levels of TNF-α, IL-6, IL-17A and IL-10 in paw homogenates were all measured using murine ELISA kits (eBioscience, San Diego, CA) according to the manufacturer’s instructions.

### Statistical analyses

Data were expressed as the mean ± standard deviation. Two-tailed Student’s *t*-test was used to compare intergroup differences. To compare multiple experimental groups, one-way or two-way ANOVA with Dunnett’s *post hoc* test was used (GraphPad Prism v5.0 software, La Jolla, CA). Statistical significance was set at *p* < 0.05.

## Results

### Effects of alantolactone on cell viability

In the first series of experiments, the cytotoxicity of alantolactone was quantified by LDH released against those from cultured naïve CD4 T cells of normal C57BL/6 (B6) mice. We found that cells exposed to alantolactone at a concentration of 0.5–5 μM showed viability similar to controls (Figure 1(B)). In the CCK-8 assay for assessment of the same cytotoxicity, we similarly found no viability effect on CD4 T cells (Figure 1(C)). These results indicated that treatment with alantolactone at concentrations ≤5 μM had no effect on the studied cell viability.

### Alantolactone inhibits IL-17 production by CD4 T cells under Th17-polarizing conditions

**Figure 2.**
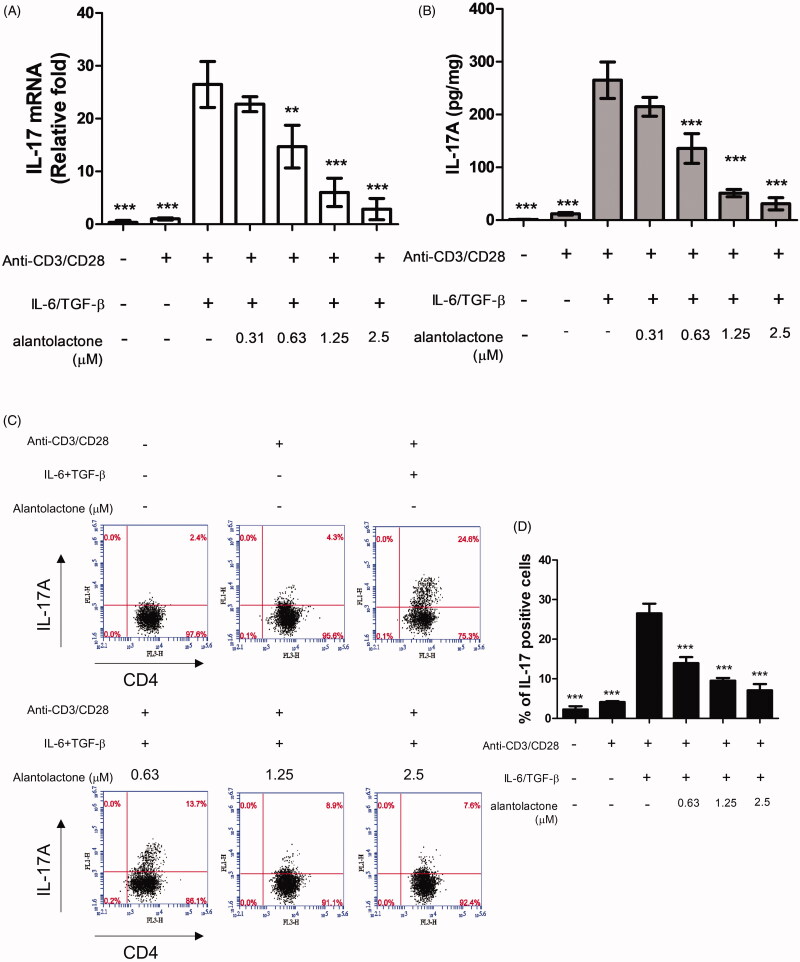
Alantolactone inhibited Th17 differentiation *in vitro*. (A) IL-17 mRNA and (B) secreted IL-17A in culture medium were analysed by real-time RT-PCR and ELISA, respectively, at 72 h. The results of real-time PCR are presented as fold changes relative to the untreated control (set as 1.0). (C) The percentage of IL-17 cells among CD4 T cells was examined by flow cytometry. (D) Data are representative of three independent experiments, and values are expressed as the mean ± SEM of samples from three wells. **p < 0.01, ***p < 0.001 compared with CD4 T cells under Th17-polarizing conditions without alantolactone treatment, as determined by one-way ANOVA with Dunnett’s test.

**Figure 3.**
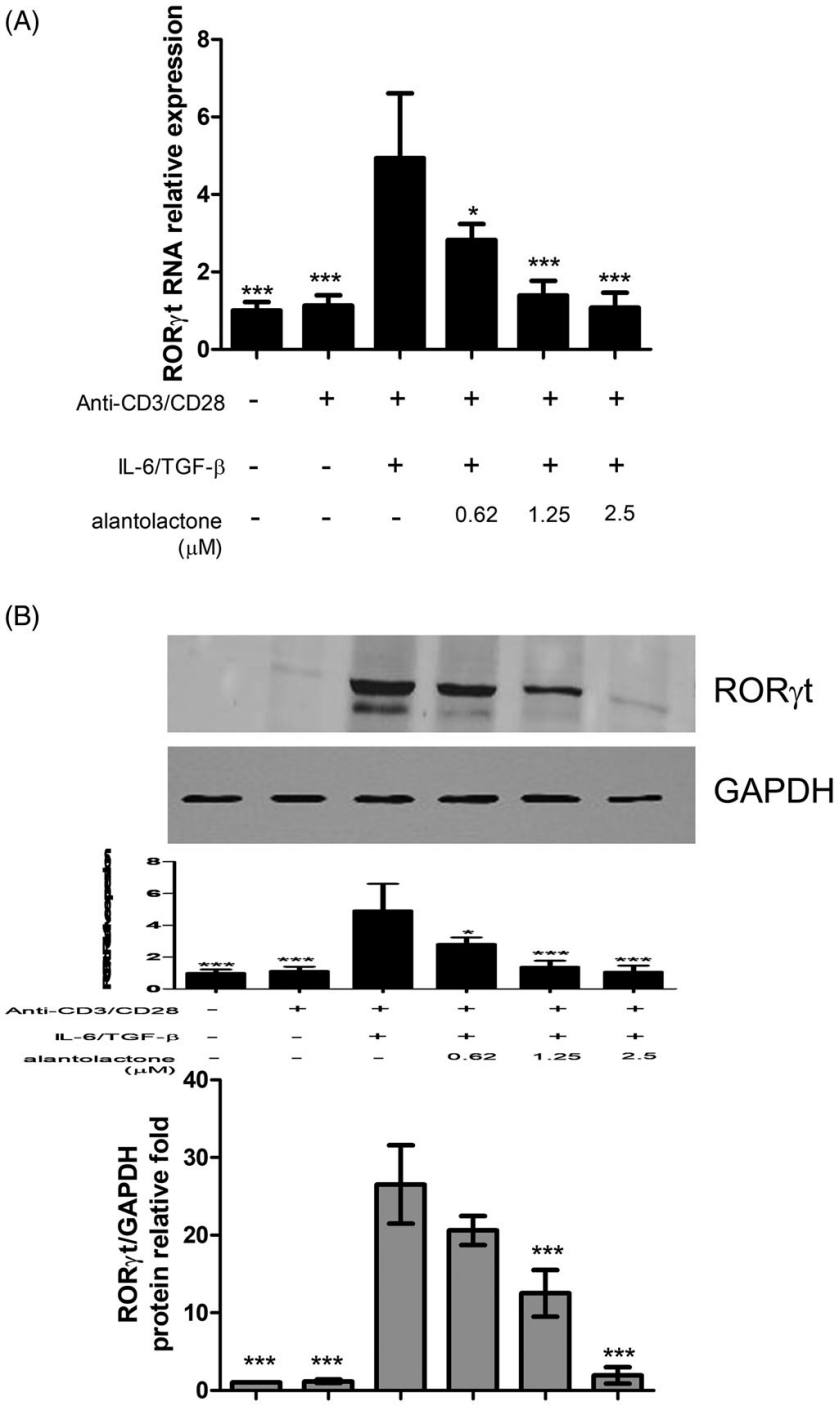
Alantolactone suppressed RORγt expression in CD4 T cells under Th17-polarizing conditions. (A) RORγt mRNA and protein expression was measured by real-time PCR and (B) Western blotting, respectively, at 72 h. Densiometric measurements of RORγt protein levels were normalized to the corresponding total protein level and expressed as relative values. Data are representative of three independent experiments, and values are expressed as the mean ± SEM of samples of three wells. *p < 0.05, ***p < 0.001 compared with CD4 T cells under Th17-polarizing conditions without alantolactone treatment, as determined by Student’s *t*-test.

To study the drug effects on the differentiation of Th17 cells, we initially analysed the mRNA expression and protein secretion of IL-17 in Th17-polarized CD4 T cells in C57BL/6 mice. The results showed that IL-17 mRNA expression in these cells was higher than that in untreated controls, while alantolactone suppressed, in a dose-dependent manner, the increased mRNA expression (Figure 2(A)) and levels of IL-17 secreted in the medium (Figure 2(B)). In addition, the percentages of CD4^+^ IL-17^+^ T cells in the population were reduced by alantolactone in a dose-dependent manner (Figure 2(D)). Moreover, alantolactone suppressed the increased expression of RORγt under Th17-polarizing conditions (Figure 3(A,B)). Taken together, the results are consistent with an effect of alantolactone in suppressing *de novo* Th17 cell differentiation from naïve T cells.

### Alantolactone treatment inhibits STAT3-signalling

**Figure 4.**
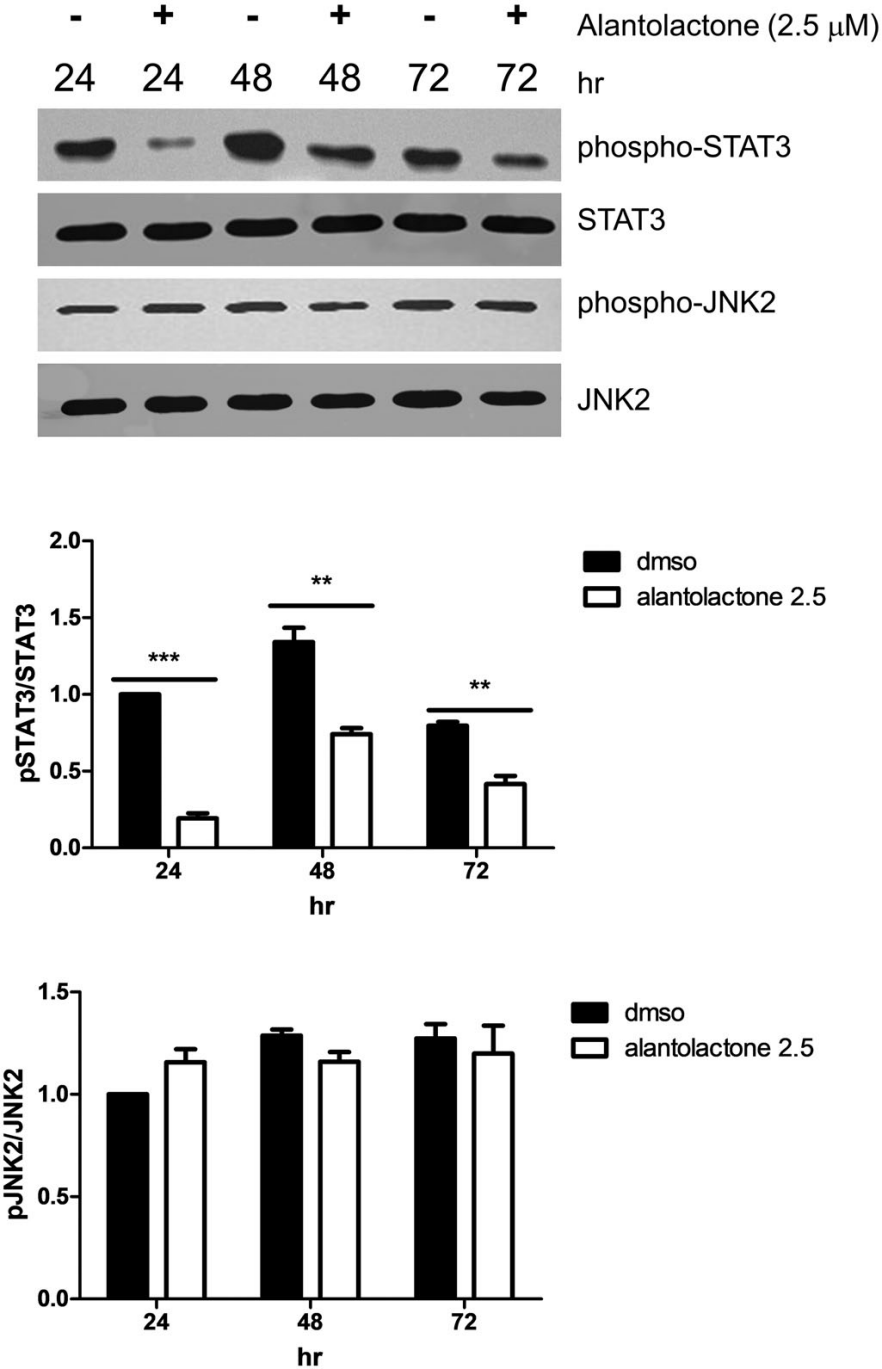
Alantolactone inhibited STAT3 phosphorylation in Th17-polarizing CD4 T cells at 24, 48 and 72 h. The expression levels of STAT3 and JAK2 with and without phosphorylation were determined by Western blotting. Data are expressed as relative results using ImageJ software compared with CD4 T cells under Th17-polarizing conditions without alantolactone treatments. Data are representative of three independent experiments, and values are expressed as the mean ± SEM of samples of three wells. **p < 0.01, ***p < 0.001 compared with CD4 T cells under Th17-polarizing conditions without alantolactone treatment, as determined by Student’s *t*-test.

IL-6-mediated JAK2/STAT3 signalling is known to be important in Th17 cell differentiation (Wang et al. 2009). Therefore, we investigated the alantolactone effect on the phosphorylation of JAK2/STAT3 (pSTAT3, Tyr705 and pJAK2, Tyr1007). Protein expression of both pSTAT3 and pJAK2 is upregulated in Th17-polarizing CD4 T cells. Here, we found that alantolactone significantly inhibited STAT3 phosphorylation. However, it had no effects on the expression of JAK2 (Figure 4). The results suggested that in Th17 differentiation, alantolactone inhibits STAT3 phosphorylation independent of JAK2.

### Alantolactone reduces the severity of CIA in DBA/1 mice

**Figure 5.**
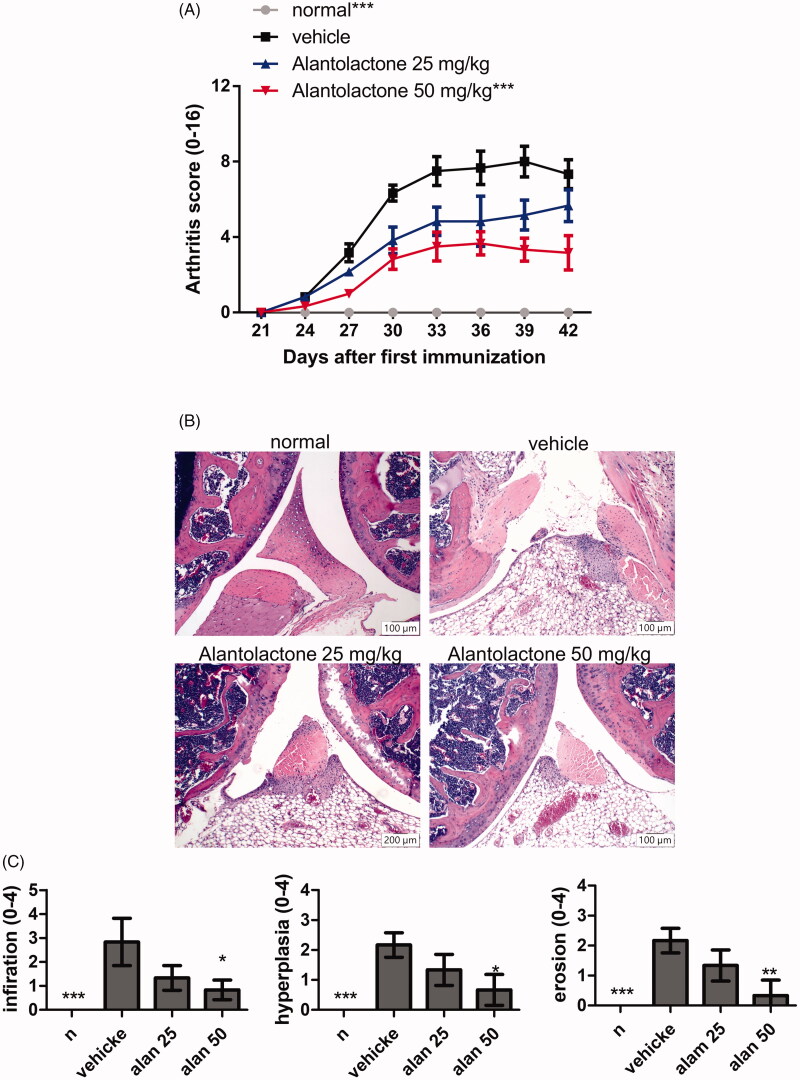
Alantolactone ameliorated the severity of CIA in mice. Beginning the day after the booster immunization, alantolactone (25 and 50 mg/kg) or vehicle (10% DMSO and 90% glyceryl trioctanoate) was administered orally once daily from day 21 to day 42. (A) Arthritis scores were monitored every three days after the immunization booster. Data are representative of three independent experiments, and values are expressed as the mean ± SEM (*n* = 6 per group). ***p < 0.001 versus vehicle-treated CIA control mice calculated with two-way ANOVA. (B) Histopathology of arthritic joints of alantolactone-treated and vehicle-treated mice by H&E staining of representative sections and (C) by histology scores at day 42 postimmunization (original magnification ×100). *p < 0.05, **p < 0.01, ***p < 0.001, versus the CIA vehicle group, as determined by one-way ANOVA with Dunnett’s test.

Given the finding that alantolactone inhibits Th17 differentiation, we further investigated the related immune regulatory properties. Immunization with a bovine CII emulsion in DBA/1 mice led to arthritis. Arthritis was characterized by paw swelling, erythema, oedema and joint rigidity. These symptoms were all alleviated under oral administration of alantolactone (25 and 50 mg/kg) daily for two weeks (Figure 5(A)). To further determine the severity of arthritis, we performed a histology study on the ankle joint on day 42. Consistent with our visual observations of symptomatic relief, alantolactone-treated mice showed significant reductions in the following histological features: synovial hyperplasia, infiltrating inflammatory cells, cartilage destruction and bone erosion in the ankle joints (Figure 5(B,C)).

### Inflammatory cytokines were suppressed by alantolactone administration

**Figure 6.**
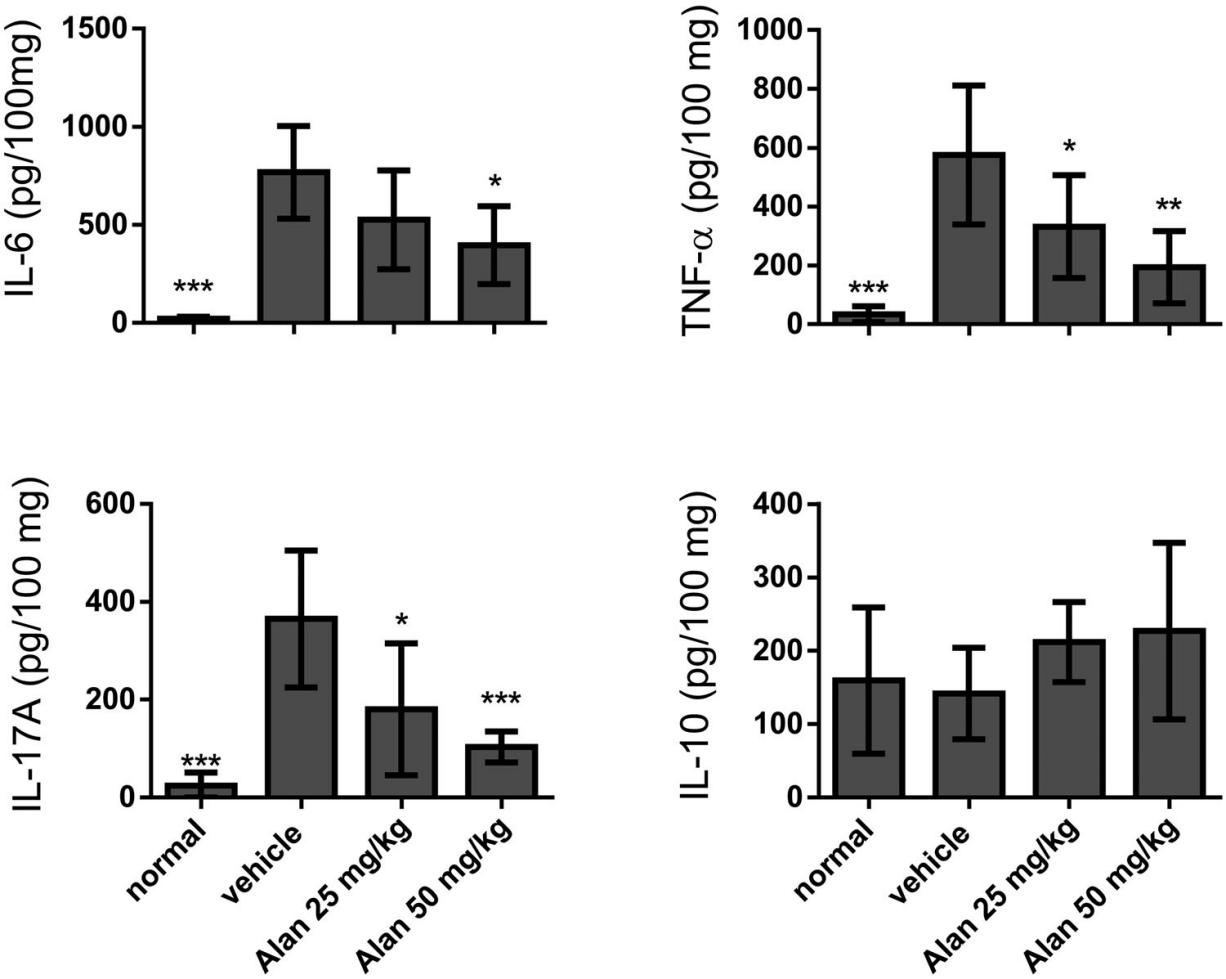
Evaluation of cytokine production in CIA mouse homogenized paw tissues under crotonoside treatments. The expression levels of the proinflammatory cytokines IL-6, TNF-α and IL-17A and the anti-inflammatory cytokine IL-10 were determined by ELISA for each tissue sample. Data (*n* = 6 per group) are representative of three experiments. Bar graphs are representative of three independent experiments. Values are expressed as the mean ± SEM (*n* = 6 per group). *p < 0.05, **p < 0.01, ***p < 0.001, versus the CIA vehicle group, as determined by one-way ANOVA with Dunnett’s test.

To further study the microenvironment of the mouse joint synovium and the anti-arthritic activity of alantolactone, we measured local levels of pro-inflammatory cytokines, such as TNF-α, IL-6 and IL-17A, as well as the anti-inflammatory cytokine IL-10, by ELISA in paw tissues of CIA mice. We found that after alantolactone treatment, these four proinflammatory cytokines were all markedly higher following the establishment of CIA. More importantly, TNF-α, IL-6 and IL-17A were significantly reduced upon alantolactone treatment (Figure 6). At the same time, the anti-inflammatory cytokine IL-10 remained unchanged.

### Alantolactone treatment suppresses the CII-specific immune response in CIA mice

**Figure 7.**
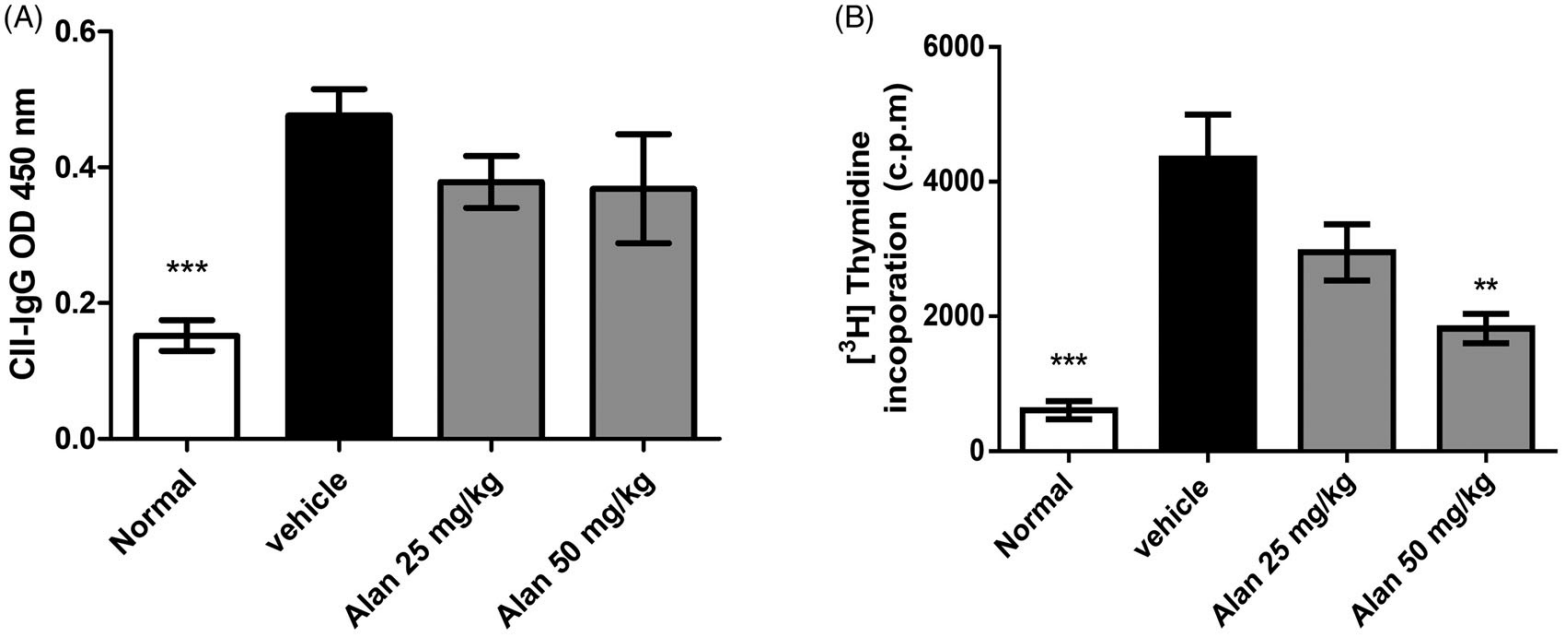
Alantolactone suppressed type II collagen (CII)-specific immune responses in mice with CIA. (A) Forty-two days postimmunization, serum was obtained from alantolactone- or vehicle-treated mice, and anti-CII IgG was measured by ELISA at a 1:2500 dilution. (B) Spleens were harvested at day 42 postimmunization and tested for their ability to proliferate *in vitro* for 72 h in medium containing CII (50 μg/mL). The proliferation of the spleen was measured by ^3^H-TdR incorporation, and the stimulation index was calculated. Bar graphs representative of three independent experiments. Values are expressed as the mean ± SEM (*n* = 6 per group). **p < 0.01, ***p < 0.001, versus the CIA vehicle group, as determined by one-way ANOVA with Dunnett’s test.

In CIA pathogenesis, both humoral and T cell responses are important (Courtenay et al. 1980). We further measured the serum titres of CII-specific antibodies and the expansion of splenic CII-specific T cells. Consistent with previous studies, we found high titres of serum anti-CII IgG in vehicle-treated CIA mice. However, IgG titres were unchanged in the alantolactone-treated CIA mice (Figure 7(A)). In addition, spleen cells obtained from both vehicle-treated and alantolactone-treated CIA mice on day 42 were stimulated with CII *in vitro*. Spleen cells showed less proliferation in alantolactone-treated CIA mice than in vehicle-treated controls (Figure 7(B)).

### Alantolactone decreases the percentage of Th17 cells in the spleens of CIA mice

**Figure 8.**
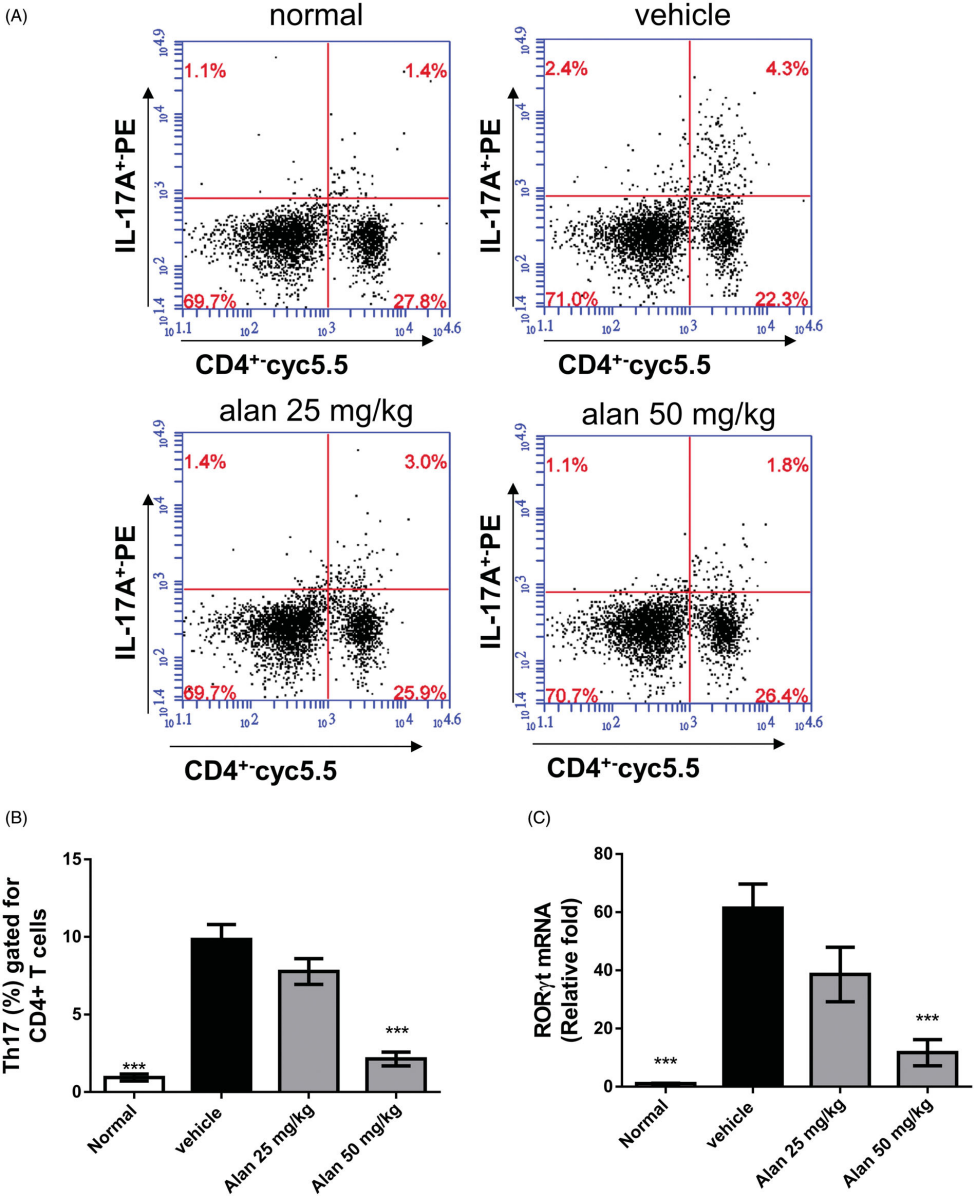
Alantolactone suppressed the Th17 immune response in mice with CIA. Splenocytes were obtained on day 42, cultured with CII (50 μg/mL) for 72 h, (A) stained with anti-CD4-Prep and anti-IL-17 17A-FITC antibodies, and analysed by flow cytometry. (B) Bar plots of the mean ± SEM from one of three independent experiments each with three or four mice/group. (C) The expression of ROR-γT mRNA in mouse spleen. Splenocytes were harvested on day 42, and relative expression levels of mRNA were measured by quantitative real-time RT-PCR using the ΔΔCT method with GAPDH mRNA as an internal control. Bar graphs representative of three independent experiments. Values are expressed as the mean ± SEM (*n* = 6 per group). ***p < 0.001, versus the CIA vehicle group, as determined by one-way ANOVA with Dunnett’s test.

Th17 cells are known to play an important role in the pathogenesis of RA (Courtenay et al. 1980). We hypothesized that the reduced arthritic symptoms in alantolactone-treated CIA mice are the result of fewer Th17 cells. To test this hypothesis, we assessed the profiles of CD4^+^ T cell subsets in splenocytes of CIA mice. We found significant reductions in the percentage of CD4^+^IL-17A^+^ Th17 cells after alantolactone treatment compared with controls (Figure 8(A,B)). Moreover, lower RNA expression of RORγt was found in the spleens of CIA mice after alantolactone treatment (Figure 8(C)). Taken together, these findings suggested that alantolactone reduces the Th17 cell population in the spleens of CIA mice.

## Discussion

We have presented the results of the first study in a mouse model showing that alantolactone is therapeutically effective against CIA. Its beneficial effects were the alleviation of arthritic symptoms with histological improvement. These findings could be related to the anti-inflammatory properties of alantolactone, which include the suppression of B and T cell responses and downregulated joint inflammatory cytokines. Furthermore, in murine spleen cells, alantolactone suppressed Th17 polarization through STAT3 signalling. In summary, alantolactone is a potential therapeutic agent for RA.

Alantolactone, a natural compound purified from plants, exerts anti-inflammatory effects through multiple mechanisms in a variety of cells. It is known to have many actions, including the suppression of inducible nitric oxide synthase and cyclooxygenase-2 in lipopolysaccharide-stimulated macrophages (Chun et al. 2012), activation of nuclear factor kappa light chain enhancer of activated B cells (NF-κB) and inflammatory cytokine and chemokine expression in keratinocytes after stimulation with interferon (IFN)-γ and TNF-α (Lim et al. 2015) or TNF-α alone (Wang, Gao, et al. 2018), TNF-α-induced activation of NF-κB and mitogen-activated protein kinase (MAPK) pathways in endothelial cells (Gao et al. 2017), inflammatory cytokine expression in human respiratory epithelial cells (Mazor et al. 2000; Gierlikowska et al. 2020) and neutrophils (Gierlikowska et al. 2020), and inflammatory cytokine and chemokine expression in TNF-α-stimulated synovial fibroblasts (Gao et al. 2017). In addition, alantolactone likely has antioxidant properties through the induction of detoxifying enzymes (Seo et al. 2008). In view of oxidative stress occurring during inflammation (Zuo et al. 2015), the anti-inflammatory effects of alantolactone could be further enhanced. Indeed, alantolactone has known therapeutic potential against inflammatory responses in several rodent models, such as atopic dermatitis (Wang, Gao, et al. 2018) and traumatic brain injury (Wang, Lan, et al. 2018). Moreover, sesquiterpene lactones extracted from *Inula helenium*, which consist mostly of alantolactone and isoalantolactone, ameliorate the severity of arthritis during the early and later stages of arthritis in rat models (Gao et al. 2017). Our present results on CIA are in line with earlier findings.

Proinflammatory cytokines contribute to RA pathogenesis (Chabaud et al. 2001; Choy and Panayi 2001; Van Bezooijen et al. 2002; Firestein 2003; McInnes and Schett 2007). Proinflammatory cytokines, such as TNF-α and IL-6, participate in the propagation of joint inflammation in RA (Choy and Panayi 2001; Firestein 2003; McInnes and Schett 2007). In the synovium and synovial fluids of RA patients, IL-17A expression is higher (Chabaud et al. 2001; Raza et al. 2005). IL-17A can induce inflammation by promoting the production of proinflammatory cytokines, such as TNF-α, IL-1β and IL-6 (Van Bezooijen et al. 2002; Mills 2008). Moreover, injection of IL-17A into a normal knee produces joint inflammation and bone and cartilage destruction (Chabaud et al. 2001). In line with these observations, our experiments demonstrated that alantolactone dose-dependently reduced the expression of TNF-α, IL-6 and IL-17A at the inflamed joints. These results represent collective evidence that alantolactone has anti-inflammatory effects, and these effects might be therapeutic against CIA.

The importance of Th17 cells in RA pathogenesis is evident in various animal studies (Hashimoto 2017). Studies on RA patients also reported increased populations of Th17 cells in blood and bone marrow (Shen et al. 2009; Li et al. 2017), as well as higher levels of IL-17 in serum, which correlate with disease activity (Kotake et al. 1999; Ziolkowska et al. 2000; Dhaouadi et al. 2018). Clinical trials on RA patients also supported the efficacy of anti-IL-17 therapies (Kunwar et al. 2016). Based on these findings, an abnormally upregulated Th17 response contributes to the generation of RA. Although the mechanisms underlying the beneficial effects of alantolactone in CIA remain undetermined, we proposed here that the efficacy of alantolactone treatment in CIA is due to, at least in part, the suppression of the Th17 response. The evidence is listed below: (1) alantolactone administration can inhibit spleen Th17 cell expansion *in vivo* in CIA mice and (2) alantolactone can inhibit Th17 differentiation from spleen CD4+ T cells *ex vivo*. We further demonstrated that alantolactone inhibited the Th17 response in CIA mice via the suppression of STAT3 signalling, which in turn reduced RORγt expression. Our results were compatible with reported studies on tumours (Khan et al. 2013; Maryam et al. 2017; Zheng et al. 2019) and IL-6-induced muscle inflammation (Kim et al. 2017), in which STAT3 activation is inhibited after alantolactone treatment through mechanisms such as STAT3 glutathionylation (Maryam et al. 2017) and direct disruption of STAT phosphorylation (Zheng et al. 2019).

## Conclusions

Alantolactone has a beneficial effect against the development of CIA. Alantolactone exerts therapeutic effects through the suppression of inflammatory cytokines and the modulation of immune responses. With an acceptable cost-effectiveness and safety profile, add-on alantolactone treatment could be an alternative option for treating RA patients. However, its practical use in combination with DMARDs or biologics for RA requires further study.

## Data Availability

The data that support the findings of this study are available from either of the corresponding authors, Chi Chien Lin or Kuo Tung Tan, upon reasonable request.
